# Application of Visible/Infrared Spectroscopy and Hyperspectral Imaging With Machine Learning Techniques for Identifying Food Varieties and Geographical Origins

**DOI:** 10.3389/fnut.2021.680357

**Published:** 2021-06-17

**Authors:** Lei Feng, Baohua Wu, Susu Zhu, Yong He, Chu Zhang

**Affiliations:** ^1^College of Biosystems Engineering and Food Science, Zhejiang University, Hangzhou, China; ^2^Key Laboratory of Spectroscopy Sensing, Ministry of Agriculture and Rural Affairs, Hangzhou, China; ^3^School of Information Engineering, Huzhou University, Huzhou, China

**Keywords:** visible/infrared spectroscopy, hyperspectral imaging, variety, geographical origin, machine learning

## Abstract

Food quality and safety are strongly related to human health. Food quality varies with variety and geographical origin, and food fraud is becoming a threat to domestic and global markets. Visible/infrared spectroscopy and hyperspectral imaging techniques, as rapid and non-destructive analytical methods, have been widely utilized to trace food varieties and geographical origins. In this review, we outline recent research progress on identifying food varieties and geographical origins using visible/infrared spectroscopy and hyperspectral imaging with the help of machine learning techniques. The applications of visible, near-infrared, and mid-infrared spectroscopy as well as hyperspectral imaging techniques on crop food, beverage, fruits, nuts, meat, oil, and some other kinds of food are reviewed. Furthermore, existing challenges and prospects are discussed. In general, the existing machine learning techniques contribute to satisfactory classification results. Follow-up researches of food varieties and geographical origins traceability and development of real-time detection equipment are still in demand.

## Introduction

Food quality and safety have aroused increasing attention. The inner quality of agricultural food is directly related to its variety and geographical origin. Different varieties of one agricultural product have considerable differences in their nutrition compounds and elements (1, 2). Geographical origins always differ in climate, soil, and agricultural practices, which have a strong influence on the chemical markers of plants (3, 4). Moreover, some products can only grow well in certain areas, which will have higher commercial values than those produced in other areas (5, 6). In addition to quality, the commercial price of food is strongly related to its varieties and regions (7, 8). Some unscrupulous merchants may sell fraudulent products at the price of the authentic ones, and some counterfeit materials will even impair consumers' health (9, 10). For instance, different apple varieties can be easily mixed during harvesting and marketing. A reliable approach to discriminate different varieties of apples is needed by apple sellers (11). In addition, waxy maize contains lots of amylopectins and is widely used for direct consumption and for producing cans, cakes, feeds, and thickener, while sweet maize has a high sugar content and is mainly used for direct consumption or processed into frozen corn and canned corn (12). Except for price and nutrition, different varieties can cause difficulty for the food processing industry. For example, different varieties of cocoa have diverse chemical compositions, making it difficult for the processing industry to standardize parameters during processing (13). Coffee beans from geographical origins that are known to produce high-quality beverages have a great commercial value (5). Consequently, discrimination of varieties and regional origins will contribute to cracking down fraud, developing a steady market, and protecting consumers' health.

There are some traditional methods to discriminate food varieties and geographical origins, including detection by experienced experts, sensory analysis (14), and wet chemistry methods [high-performance liquid chromatography (HPLC) (15), Gas chromatography (GC) (16), Gas Chromatography-Mass-Spectrometry (GC-MS) (17), Proton Transfer Reaction-Mass Spectrometry (PTR-MS) (17) and stable isotopic analysis (18)]. Detection by experienced experts is straightforward, but it requires expertise and experience and can be subjective. Therein, sensory analysis is manipulated by organizing a certain number of volunteers or panelists to evaluate a product with their sensory system. The evaluation indexes include sensory appearance, smell, flavor, and taste (14). These wet chemistry methods are precise to detect almost all components in agricultural products. However, all of them usually consume too much time and require a large amount of reagent, and the operation process is complicated. These methods are destructive methods.

Since spectra contain chemical information of food, it could reflect the distinction among food spectra from different varieties and geographical origins. Thus, spectra can be exploited to trace varieties and regions of food. Therefore, spectroscopic approaches, such as visible/near-infrared spectroscopy (VIS/NIR), near-infrared (NIR), mid-infrared spectroscopy (MIR), and hyperspectral imaging (HSI), have been widely used in the analysis of agricultural products. HSI can provide spectral information and spatial one simultaneously, which has been popular for varieties and regions discrimination of food (19, 20).

To further promote the development of researches and practical applications of traceability of food varieties and geographical origins, a system review outlining the progress of related studies and corresponding analytical methods is in demand. This work is the first to provide a systematic overview of the applications of visible/infrared spectroscopy and hyperspectral imaging technologies in identifying food varieties and geographical origins with machine learning methods. Classical and novel machine learning methods for feature selection/extraction and modeling in identifying food varieties and geographical origins are also reviewed. Furthermore, this review has proposed the existing problems and potential ways to deal with them.

## Brief Introduction of VIS/IR and Hyperspectral Imaging

### VIS/IR

VIS is an electromagnetic spectrum at the spectral range of 380 to 780 nm, providing color information for food classification. NIR and MIR spectroscopies are different regions of infrared spectroscopy. The NIR region extends from 780 to 2,500 nm, between the VIS and MIR (from 2,500 to 15,000 nm) (21). In general, a typical NIR system consists of four components, including a light source, light-isolating mechanisms, detectors, and sampling devices., which was introduced in detail in (22). The basic principle of NIR and MIR is that chemical bonds such as C-H, N-H, O-H, and S-H bonds can absorb infrared radiation at specific wavenumbers, which correspond to different characteristic peaks or valleys (23). Consequently, the chemical components of the samples can be verified by extracting the relevant information from the spectral profiles with chemometric methods. Contrast to some wet chemistry approaches, NIR is faster, more convenient and non-destructive, and has been widely used for qualitative and quantitative analyses (9, 24–28).

The MIR spectrum has a higher specificity than the NIR spectrum, and it is considered to be more appropriate for identification and characterization purposes (29, 30). A comparison between MIR and NIR can be found in this work (29). MIR can be used to detect compositional differences between food samples based on vibrations of various chemical groups at specific wavelengths in the mid-infrared range (29). The information provided by using these fundamental absorption bands of MIR can proffer information regarding the chemical structure of a food sample.

### Hyperspectral Imaging

Hyperspectral imaging obtains images at continuous wavebands over a specific spectral region. This emerging spectroscopic technology combines the advantages of spectroscopy and imaging, which can provide both spectral and spatial information. A typical HSI system comprises the following components: a light source, a wavelength dispersion device (spectrograph), an area detector (camera), a translation stage, and a computer (31, 32). The spectral signature obtained from HSI is unique as it results from the physical and chemical properties of the particular material measured (33). The 3D hyperspectral image cubes [I(x, y, λ)] can be obtained by four approaches, including two spatial scanning methods (point scanning and line scanning) and two spectral scanning methods (area and plane scanning) (31). The pros and cons of different scanning methods were introduced in (34). Among these methods, line scanning is the most popular method to acquire hyperspectral images for food classification.

## Summary of Identification of Food Varieties and Geographical Origins With the VIS/IR and HSI

In this review, the applications of VIS/IR and HSI are separately summarized in section Applications of VIS/IR to Trace Food Varieties and Geographical Origins and Application of Hyperspectral Imaging to Trace Food Varieties and Geographical Origins, including common crop food (rice, wheat, maize, and barley malt), beverage (tea, coffee, and chrysanthemum), fruits (grape, apple, sugarcane, loquat, mandarin, strawberry, lychee, and nectarine), nuts, meat, edible oil and other application (such as honey, *Auricularia auricular*, Chinese quince, okra kernels, and mung beans). The sample preparation, the analyzed spectral range, the signal mode, the spectral preprocessing methods, the feature selection/extraction methods, and the classification algorithms arefurther summarized in this section. The difference between VIS/IR and HSI and the corresponding different data analysis methods are discussed.

### Sample Preparation and Equipment Setting for Food Classification

Overall, there were some factors to be considered for both two technologies when preparing samples, such as the form of the sample and the total numbers of the samples. According to the summarization of VIS/IR and HSI applications, the form of crop food has an influence on the performance of classifiers. The influence of the single seed and the flour of seed has been investigated and compared (35–37). Besides, the bulk mode outperformed the single seed mode to classify maize seed varieties (38). Moreover, the harvest year influenced the varieties and geographical origins classification as well. For the relatively bigger size of samples like fruits, the sample size has a noticeable effect on spectra acquisition, thus influenced the effectiveness of classification models (39, 40). Furthermore, the performance of classifiers could vary with the number of training samples (19, 41). In particular, a small sample size could limit the ability of CNN. Careful preparation and splitting of samples were necessary to develop a more generalized model.

Since both VIS/IR and HSI were capable of collecting spectral information, they had a common criterion to select spectral range. In terms of the spectral range, different spectral ranges were adopted for classification with the same purpose, and all obtained satisfactory results. Therefore, the spectral ranges could be selected according to the practical condition. For example, the 833–2,500 nm, 908–1,672 nm, and 1,000–2,500 nm were adopted by Cui et al. (38), Yu et al. (42), and Qiu et al. (43), respectively, and all achieved the accuracy of over 97% for maize varieties classification. There existed differences regarding spectra information collection between VIS/IR and HSI. Generally, VIS/IR collects spectra through point scanning and usually measures samples several times. Thus, spectral information was limited to a relatively small sample area by VIS/IR, while HSI provided larger scanned areas (44). HSI collects hyperspectral images through four approaches mentioned in Section Hyperspectral Imaging Hyperspectral imaging. Comparing to that VIS/IR analyzes the sample in bulk and determines an average composition across the entire sample, HSI has the advantage that it provided spatial distribution of quality parameters of samples (45).

In terms of the signal mode of spectroscopic techniques, the reflectance mode is the most frequently used signal mode among three different spectra acquisition modes (reflectance, transmittance, and interactance). The reason can be that the reflectance mode effectively collects the information related to the inner quality of samples and external physical properties (such as shape and texture) and is easy to operate. The transmittance mode is usually utilized to detect liquid samples like fruit juice and relative transparent materials such as fish and fruit. The transmittance mode has the limitation that the signal is easily affected by the thickness of samples (31). The interactance mode has fewer surface effects than the reflectance mode and reduces the influence of thickness compared to transmission mode (31). Therefore, the selection of spectral mode is not compulsory, and it depends on the practical condition.

Regarding the implementation process (in-line, off-line), the majority of researchers conducted an off-line classification. Cortés et al. developed an in-line VIS/NIR spectroscopy prototype for in-line identification of five apple varieties, achieving the accuracy of 98% and 85% for red and yellow apple varieties (40). This work revealed that in-line application needed to solve the variant problem of sample shape. In this work, the problem was solved by automatically moving the probe to keep the same distance between the probe and the samples regardless of their size. Therefore, when developing an in-line application, it is essential to consider the way to obtain spectra and the rapidness and robustness of the algorithm.

### Summary of Machine Learning Methods for Food Classification

**Figure 1:**
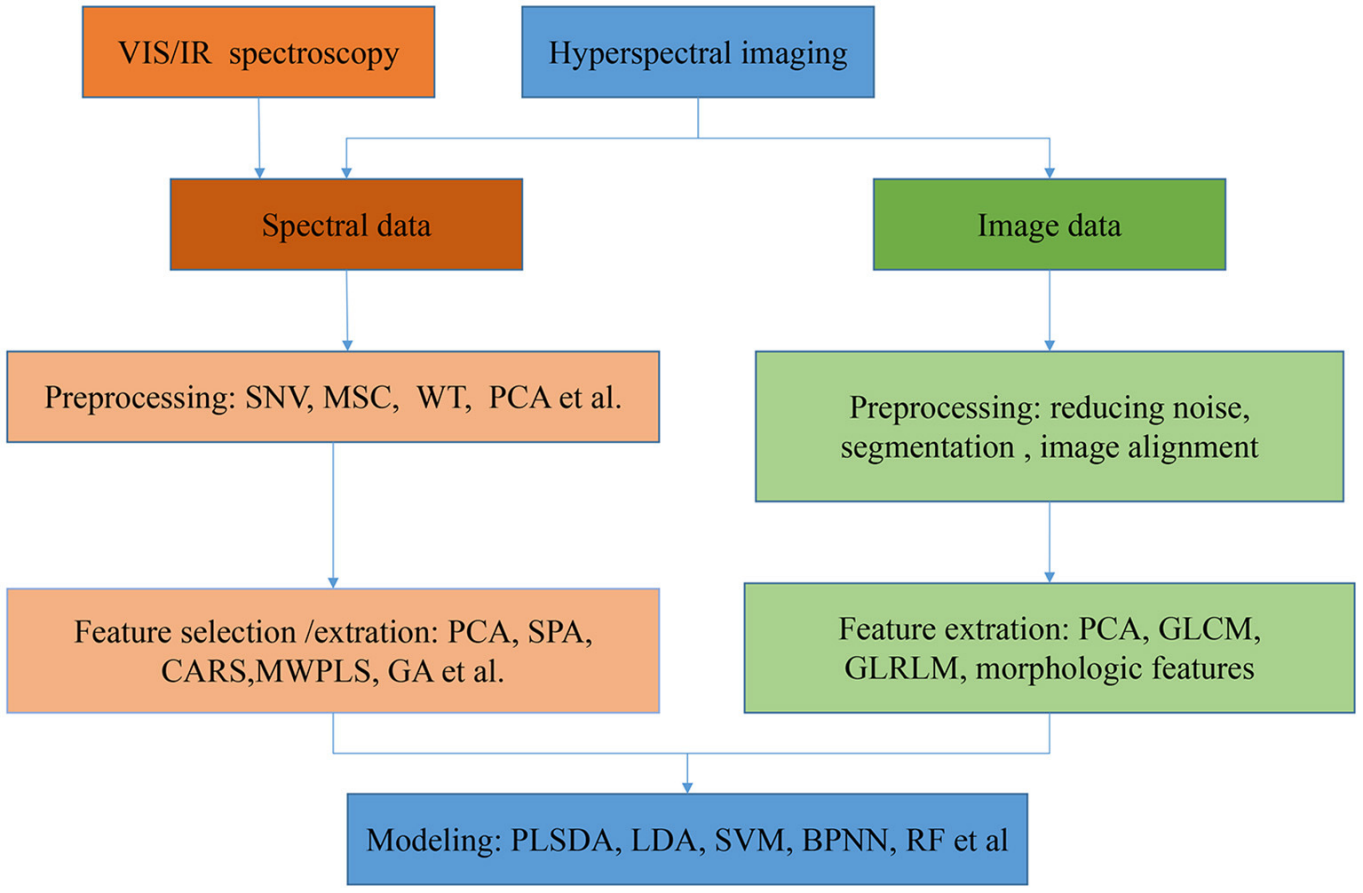
The general flowchart of procedures for food varieties and geographical origins identification model establishment.

The procedures to establish classification models with VIS/NIR, NIR, MIR spectroscopies, and HSI are shown in Figure 1. Overall, it was nearly the same to preprocess the spectra and build models based on spectra with both VIS/IR and HSI. But image features of HSI can also set as a part of features for building discriminative models. HSI is a prevalent and promising technique for food classification since it can offer both spectral and spatial information. Currently, the spectral range used by HSI is mainly in the VIS/NIR range, with few studies with HSI in the MIR range.

Before applying models for classification, it is essential to preprocess the spectra to eliminate the noises, scatter effect, and baseline shift for VIS/IR and HSI. Some works compared several different pre-processing methods before establishing classifiers (46–48). However, we contend there is no need to write the process of comparing the preprocessing in the paper because these methods are already common. Therefore, the comparison process can be removed, and optimal preprocessing can be directly adopted. The pre-treatments contain smoothing, scatter and baseline correction and derivation methods. Generally, there are several smoothing approaches to eliminating noises existing in the spectra, including Savitzky-Golay (S-G) smoothing algorithm (the 1st and the 2nd derivative), moving smoothing, wavelet transform (WT), and Karl Norris derivative filter (KND). At present, the S-G smoothing is the most widely used method to process spectra (46). Besides, MSC, SNV, OSC, DOSC, and de-trending are commonly used to correct addictive and multiplicative effects in the spectra. These different pre-treatments can be used individually or in combination. The preprocessing methods are universal regardless of the type of spectrum. As most researches were conducted in the laboratory, the environmental conditions were well-controlled, but there was more uncertainty in the practical application situation. Thus, it requires selecting a proper preprocessing method according to the characteristic of samples and the detection environment. In a nutshell, there are no specific criteria for selecting the spectral preprocessing method. It should be determined according to the practical application situation, and different combinations of these preprocessing methods may improve the performance of the model.

Given both VIS/IR and HSI containing numerous wavelengths, feature extraction and feature selection methods are utilized to reduce the data dimension and develop a more simple and accurate model. These feature extraction and feature selection methods are common to both VIS/IR and HSI. PCA was the most widely used feature extraction method, powerful in reducing data dimension while maintaining the information in original data. Besides, LDA (38), WT (49), and a newly proposed spectral feature-extraction method based on waveform resolution (SFEWR) (50) were also applied for feature extraction. In addition, many effective wavelengths selection methods were used, including weight coefficients of PCA loadings (51, 52) and partial least-squares (PLS) (1, 53), successive projections algorithm (SPA) (54, 55), genetic algorithm (GA) (3), the 2nd derivative (51, 56), uninformative variables elimination based on partial least squares (UVE-PLS) (57, 58), competitive adaptive reweighted sampling (CARS) (58–61).

Moreover, many studies have proposed and used novel methods for feature selection when identifying food varieties and geographical origins. These methods included ordered predictor selection (OPS) (62), stepwise discriminant analysis (SDA) (63), iteratively retaining informative variables (IRIV) (64), variable iterative space shrinkage approach (VISSA) (64), joint skewness-based wavelength selection algorithm (JSWSA) (65) and the like. Except for methods for selecting effective wavelengths, several effective wavelength interval selection methods are also applied to select feature wavelengths ranges, including interval PLS (iPLS) (57), moving window PLS (MWPLS) (66), changeable size moving window partial least squares (CSMWPLS) (67), changeable size moving window PCA (CSMWPCA) (67), backward interval PLS (biPLS) (58), synergy interval PLS (Si-PLS) (65) and the like.

Regarding image features of HSI, texture features and morphologic features are usually employed for varieties and origins classification. Gray-level co-occurrence matrix (GLCM) and gray level run-length matrix (GLRLM) are usually adopted to extract texture features from the gray-level images in each selected characteristic wavelength to avoid redundant information and the computing complexity (68).

In the food classification field, the commonly adopted supervised learning algorithms contain PLS-DA (68, 69), SVM (43, 70), LSSVM (53), RF (71), BPNN, RBFNN (19, 52), extreme learning machine (ELM) (54) and newly introduced deep convolution neural network (DCNN) (72). Among these algorithms mentioned above, PLS-DA is one of the most widely implemented chemometric methods in VIS/IR spectroscopy analysis for the advantage of handling data with collinearity. PLS-DA is a linear supervised classification method based on the PLS algorithm. The categories of samples are dummy variables with only zero and one, and the cutoff usually be set as 0.5 (73). Besides, SVM is also a commonly used classification method, which can map the original data into higher dimensional spaces with kernel function, and it optimizes a hyperplane with an appropriate margin to classify different groups (43). Radial basis function (RBF) is a usually used kernel within SVM. The two parameters within SVM (RBF kernel), penalty coefficient (C) and kernel parameter (γ), need to be optimized by a method, such as the commonly used grid-search procedure. In addition to a single model, an ensemble model such as RF is also widely used for classification. RF consists of many different decision trees that are grown from bootstrap samples of response variables. Each tree makes a vote to classify samples, and the final classification result is made according to the majority vote (71). In addition, BPNN is one of the most used neural networks for classification with IR and HSI techniques. The structure of BPNN commonly includes an input layer, a hidden layer, and an output layer. The transfer function most used between different layers is the sigmoid function. Moreover, as a deep learning method, CNN can automatically extract abstract shallow and deep features of the input, which is currently popular and a very promising tool for food classification. A typical CNN comprises multiple convolution layers (to learn abstract features) and several fully-connected layers (to realize classification of each sample) (72).

Among the unsupervised learning methods, PCA is the most popular algorithm in this field with the advantages of dimensionality reduction and visualization. PCA aims to maximize the internal information of the data after transformation and to measure the importance of the direction by measuring the variance of the data in the direction of the projection (74). The first few principal components (PCs) resulting from PCA contain the majority of information of original data and are commonly used for classification (50). Besides, there were some other unsupervised methods that were less used for classification, including hierarchical cluster analysis (HCA) (75), and some useful clustering methods such as Gustafson–Kessel (GK) clustering (76), fuzzy c-means clustering (FCM) (76, 77), fuzzy discriminant c-means clustering (FDCM)(76) and possibilistic c-means (PCM) clustering (76).

**Figure 2:**
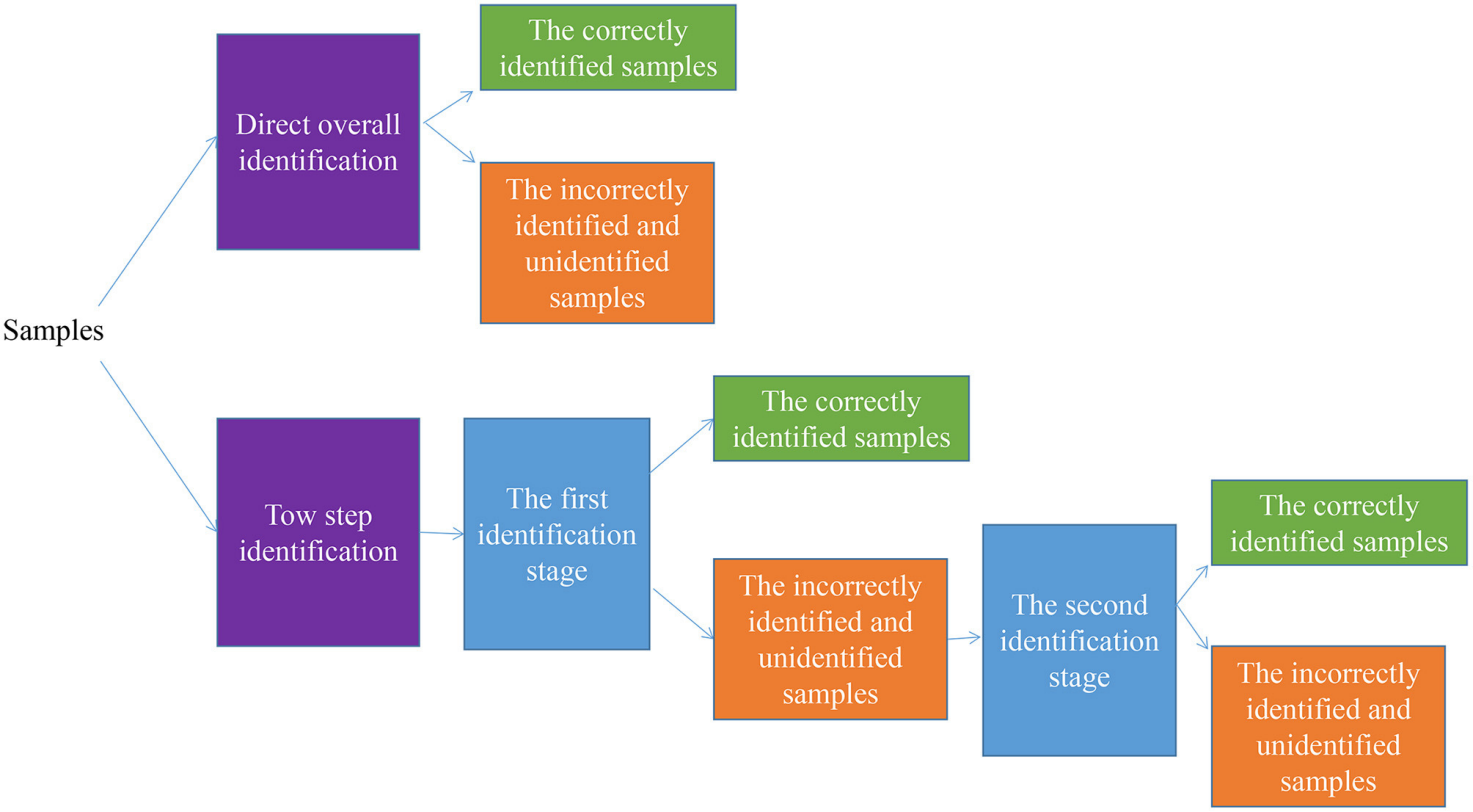
The strategy for food varieties and geographical regions classification tasks.

To handle the task of classifying the varieties or geographical origins of the food, it is common to train a model to classify samples directly. However, some strategies can be used to obtain more precise classification results. The two-step analysis can be used for classification, as shown in Figure 2. The second identification stage is applied to further classify the incorrectly identified and unidentified samples at the first identification stage.

**Figure 3:**
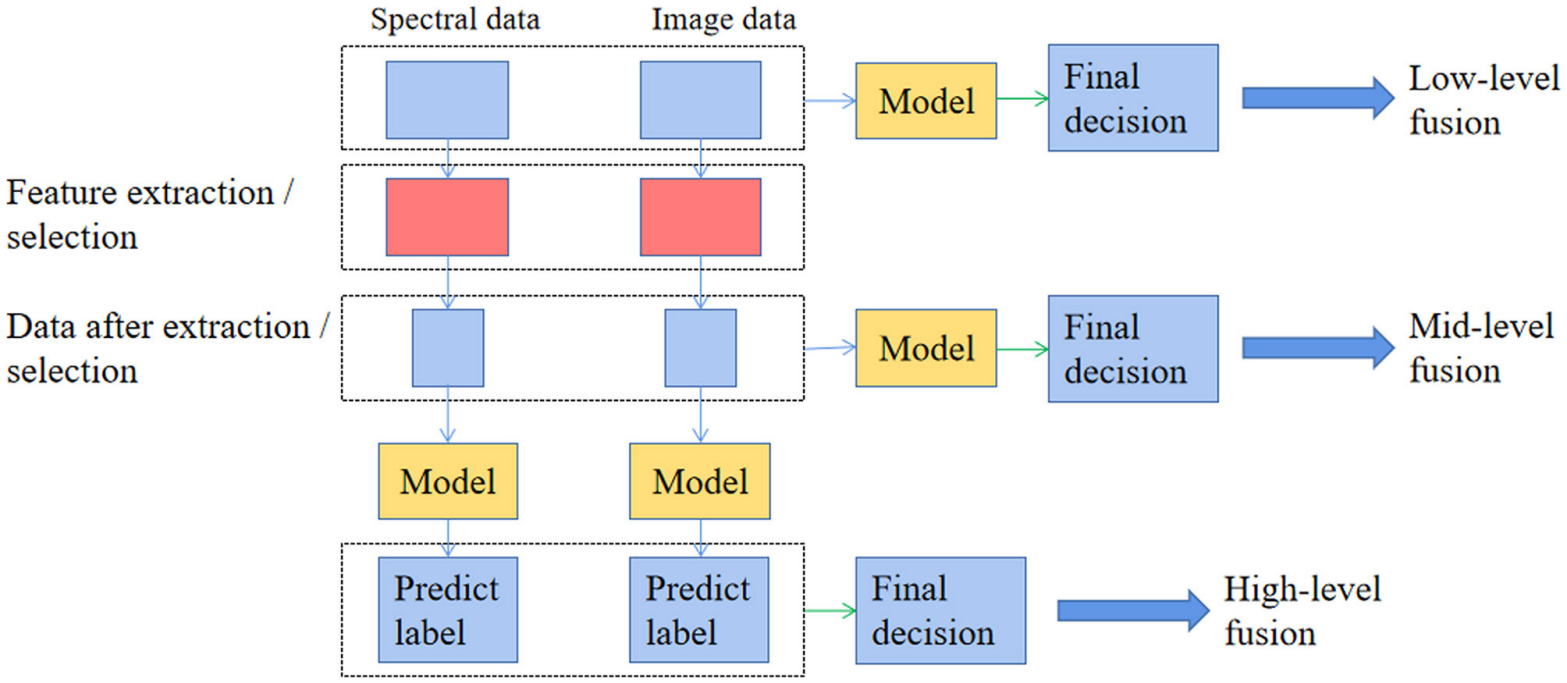
The different types of data fusion with hyperspectral imaging for food varieties and geographical origins identification.

In contrast to VIS/IR, HSI can provide both spectral and image information localized in the image domain. Therefore, a proper data fusion strategy can be considered to improve classification accuracy when applying supervised and unsupervised algorithms for classification when using HSI. According to most researches using the HSI technique, there exist three different levels of data fusion (Figure 3), low-level fusion (also known as pixel-level fusion, simply integrating two kinds of variables as inputs), mid-level data fusion (also known as feature level fusion, combining them after selecting characteristic variables, respectively), and high-level data fusion (also known as decision level fusion, considering the results of models built on each source of data to make the final decision) (78). Besides, when applying HSI for food classification, two approaches (pixel-wise and objective-wise) were frequently adopted to classify the varieties and origins of food (47, 52). Since HSI provides spatial information, it is easy to map the corresponding classification results into the image for visualization (72). That is a more intuitive method to display the difference of samples from different varieties or geographical origins. In a nutshell, visualization helps present the inner quality distribution of spatially non-homogeneous properties of interest in a sample.

## Applications of VIS/IR to Trace food Varieties and Geographical Origins

### Crop Food

Rice is one of the most vital foods and serves as the primary energy source for the most global population. Chen et al. (8) proposed an untargeted identification method combining NIR (4,000–10,000 cm^−1^) to classify three rice varieties. The work selected the ten most informative variables by a joint mutual information variable selection method to construct a one-class model obtaining the specificity and sensitivity of 100%. Regarding the identification of rice from two geographical origins, Lee et al. (79) constructed the random forest (RF) model based on NIR (4,000–9,000 cm^−1^) data to discriminate rice from two countries with a discrimination rate of 100%.

It was common that the spectra of wheat samples were collected from laboratory spectrometers. However, the difference between models based on industrial spectrometers and laboratory spectrometers was rarely investigated. Ziegler et al. (35) sed the two kinds of NIR spectrometer to discriminate five species of wheat kernels (298 kernels in total harvested in the same year) and established a five-class classifier for each spectrometer, respectively. In addition, this work also established a five-class classifier for flour samples (292 flour samples in total) collected by laboratory spectrometer and compared the performance with the model based on wheat kernels collected by laboratory spectrometer. The results showed the PLS-DA model based on kernels spectra collected from industrial spectrometer obtained 4% more accuracy than that with laboratory spectrometer, with accuracy over 80%. Moreover, models based on wheat flour data have better performance than models based on wheat kernels for varieties classification with laboratory NIR spectrometer. Wadood et al. (37) also compared the performance of models based on wheat kernels and flour for its geographical origins discrimination. The results revealed that LDA models based on flour had overall better performance than those based on whole kernels regarding three geographical origins classification. A three-class classifier was established for wheat flour harvested in each year (4 years in total), respectively. The accuracy based on flour from four different harvest years differed from each other.

Maize kernel was one of the most studied crop food as well. Considering the bulk kernels mode helps obtain sufficient information from different parts of different kernels, Cui et al. (38) compared these two modes (the bulk kernels and the single kernel) to discriminate the varieties of maize kernels with NIR in the range of 833–2,500 nm. The results indicated that the (biomimetic pattern recognition) BPR models based on spectra of bulk kernels obtained an average accuracy of over 99%, which was higher than models based on spectra of the single kernel (the average accuracy of 97.2%). Furthermore, unlike most researches used bare maize kernels as samples, Jia et al. (80) tried to discriminate cultivars of coated maize kernels rather than bare kernels with NIR in the range of 1,110–2,500 nm, given that kernels usually were coated with coating agents in practice. The result indicated that the coat agents increased the difficulty of the classification task. As for the progress of the classification algorithm for maize discrimination, Yu et al. (42) introduced manifold learning to distinguish haploid maize kernels from hybrid kernels with NIR in the range of 908–1,672 nm. It obtained the classification accuracy as high as 97.1%, which indicated that manifold learning was a suitable method to solve non-linear problems. Qiu et al. (43) applied a genetic algorithm (GA) to select feature wavelengths from FT-NIR (1,110–2,500 nm) to analyze sweet maize kernels from two cultivars. The results indicated that SIMCA and PLS-DA based on feature wavelengths obtained the same prediction accuracies as the best model based on full spectra. However, building classifier based on feature wavelengths saved computing time compared with building the model based on full spectra.

Porker et al. (81) employed Attenuated total reflectance (ATR) mid-infrared (MIR) spectroscopy (375–4,000 cm^−1^) combined with chemometric techniques (SIMCA, LDA, PLS-DA) to discriminate eight varieties of barley malt. It turned out that the accuracy was only 60–76% to classify six barley varieties when mixing single variety barley collected at different regions. However, a six-class classifier could obtain the accuracy of 83–100% to classify six varieties of barley collected from the same location.

The details of the references mentioned above related to crop food varieties and geographical origins are listed in Table 1.

**Table 1:** Summary of selected references for crop food classification with visible/infrared spectroscopy.

**Sample**	**Variety/** **Region classification**	**Technique**	**Spectral range**	**Mode**	**Varieties/** **Total sample numbers**	**Extraction/** **Selection method**	**Model**	**References**
Rice	Variety	NIR	4,000–10,000 cm^−1^	Reflectance	6/144	The joint mutual information-based algorithm	One-class model	(82)
Rice	Region	NIR	9,000–4,000 cm^−1^.	Reflectance	2/60	No	PCA-LDA, PLS-DA, RF	(79)
Wheat	Variety	Industrial NIR, a laboratory FT-NIR	1,200–2,400 nm, 650- 2,500 nm	Reflectance	15/1,523	No	PLS-DA	(35)
Wheat	Region	NIR	950–1,650 nm	Reflectance	3/278	No	LDA	(37)
Maize	Variety	FT-NIR	833–2,500 nm	Diffuse reflectance	42/6,769	No	BPR	(38)
Maize	Variety	FT-NIR	1,000–2,500 nm	Diffuse reflectance	2/760	GA	KNN, SIMCA, PLS-DA, SVM	(43)
Maize haploid kernels	Variety	NIR	9,08.1–1,672.2 nm	Diffuse transmission	2/200	PCA, OLDA, PCA-OLDA, LPP, SVSLPP, SVSKLPP, KLPP, Isomap, LLE, LE, LTSA	SVM	(42)
Coated maize kernels	Variety	NIR	1,110–2,500 nm	Diffuse reflectance	4/160	PCA	SIMCA, BPR	(80)
Barely malt	Variety	MIR	375–4,000 cm^−1^	Reflectance	8/162	No	LDA, PLS-DA, SIMCA	(81)

### Beverage

Green tea, black tea, oolong tea, white tea, and albino tea have been widely analyzed during the past few years. Chen et al. (46) compared the effectiveness of six different preprocessing methods and their combination to classify white tea and albino tea with NIR (4,000–12,400 cm^−1^). The results showed that the (discriminant analysis) DA model with standard normal transformation (SNV) and Karl Norris derivative filter (KND) pre-treatments obtained the highest accuracy of 100%. Furthermore, the gene expression programming projection discriminant analysis algorithm (83), boosting partial least-squares discriminant analysis (BPLS-DA) (66) and allied Gustafson-Kessel clustering (77) were proposed to discriminate tea varieties with NIR, which all could obtain decent results.

As for geographical origins classification of tea, Diniz et al. (84) constructed LDA based on effective wavelengths selected by SPA to classify five classes of tea from different geographical origins and varieties, including Argentinean green tea, Brazilian green tea, Argentinean black tea, Brazilian black tea, and Sri Lankan black tea. This five-class classifier obtained the accuracy of 100% with NIR spectra (3,800–14,000 cm^−1^). Given that the imbalanced datasets are easily occurring during sample preparation, Hong et al. (4) set macro average recall (MAR) as the criterion to evaluate the performances of classifiers based on NIR (4,000–12,000 cm^−1^). All classifiers, including LDA, SVM, SGD, DT, RF, AdaBoost, and (multilayer perceptron) MLP, obtained MAR value over 80%, and LDA and MLP achieved MAR over 93% for discriminating tea from two geographical origins. Besides, He et al. (7) proposed a two-step identification to discriminate tea from four regions. The final accuracy for the calibration set and the validation set was 98.43 and 96.84%, respectively. Fu et al. (85) proposed PLS-DA-softmax with Gaussian kernel transformation, which obtained the accuracy of 92.99% for classifying tea from 25 regions. Besides, the proposed ensemble strategy (ES)-PLS-DA achieved the highest accuracy of 93.77%. Both PLS-DA-softmax and ES-PLS-DA were superior to one-over-rest and one-over-one strategies. Zhuang et al. (86) proposed multi-wavelength statistical discriminant analysis for tea regional origins discrimination (two regions) based on NIR (1,050–2,500 nm), with the classification accuracy of 100 and 98.33% for the calibration set and the validation set.

Esteban-Diez et al. (87) employed NIR (1,110–2,500 nm) to discriminate Arabica and Robusta coffee. The results showed potential functions method with orthogonal signal correction (OSC) or direct orthogonal signal correction (DOSC) preprocessing method could obtain the accuracy of 100%. Esteban-Diez et al. (88) designed a three-class model to classify the two coffee varieties and their blends and a five-class model to classify the two pure varieties and three different blend levels of robust contents. The results indicated combining NIR (1,100–2,500 nm) with an orthogonal signal correction (OSC) pre-treatment could achieve the accuracy higher than 98% using the potential functions method.

Unlike most researches focusing on roasted coffee rather than raw coffee beans, Bona et al. (6) and Okubo et al. (89) focused on the discrimination of the geographical origin of green coffee beans. In particular, the results in Bona et al. (6) indicated NIR was superior to MIR for the geographic identification of tea.

### Fruits

Grapevine varietal classification has aroused much interest because it offers new trends in vineyard monitoring and grape quality control. Gutiérrez et al. (82) adopted an integrated portable NIR spectral analyzer (1,600–2,400 nm) to measure grapevine leaves under field conditions directly. This research compared the models based on leaves (20 leaves per variety) from 20 varieties (called a site-specific model) and the models based on leaves (total sample size 144, six varieties per vineyards, eight leaves per variety) from three different vineyards (called a global model). The results showed that the highest accuracy of the site-specific model was 87.25%, while that of the global model was 77.08%.

NIR combined with chemometric methods was successfully applied for varieties discrimination of grape products such as grape juice (90) and grape wine (91). Cozzolino et al. (90) compared the performance with MIR (375–4,000 cm^−1^) and VIS/NIR (400–2,500 nm) for discriminate two varieties of grape juice, and LDA achieved the accuracy of 86 and 80% using MIR and VIS/NIR, respectively. Besides, FI-MIR (400–4,000 cm^−1^) outperformed the FT-NIR (12,800–4,000 cm^−1^) for classifying the geographical origins of Cabernet Sauvignon wines (92).

As for the varieties discrimination of apples, both back propagation neural network (BPNN) with the preprocessing of WT (49) and moving window partial least squares discriminate analysis (MWPLSDA) (93) achieved accuracy over 96% for classifying three varieties and four varieties of apple, respectively. Wu et al. (76) proposed a novel fuzzy clustering model called fuzzy discriminant c-means clustering (FDCM clustering) to discriminate four apple varieties with NIR (3,856–4,000 cm^−1^) with the accuracy of 97%. Furthermore, the influence of variable selection algorithms on models' performance was investigated. Li et al. (94) compared the performances of PCA and SPA variable selection methods in three apple varieties discrimination with NIR (400–1,021 nm). The results indicated that ELM based on both PCA (the accuracy of 92.05%) was superior to ELM based on SPA (the accuracy of 96.67%). Apple juice as a product of apples has also been investigated. Reid et al. (95) employed MIR (800–4,000 cm^−1^) and NIR (400–2,498 nm) to analyze apple juice made of four varieties of apples, and the results showed PLS based on MIR and PLS based on NIR had a similar result.

Compared with apple variety discrimination, there exists relatively less work for apple origins discrimination. Li et al. (94) employed NIR (400–2,498 nm) for two apple geographical origins discrimination. The results showed that the ELM model had the best performance, followed by SVM and BPNN.

Sugarcane contains a large amount of sugar and is a renewable energy source of biofuel, and the classification of sugarcane varieties contributes to the sugarcane breeding program. Steidle et al. (96) used the VIS/NIR spectroscopy (450–1,000 nm) to measure spectral reflectance in the center of each sugarcane stalk divided area for four sugarcane varieties discrimination. PLS-DA, FDA, and SFDA using full spectra obtained the classification accuracy of 82, 81, and 74%, respectively.

Considering the effect of variation of cultivar or origin on the model performance, Fu et al. (97) separately constructed seven different models for the classification of loquat samples from four different origins with the same variety, samples from four different varieties with the same geographical origin, etc. Probabilistic neural networks (PNN) for discriminating the variety of samples from the same region outperformed PNN for discriminating the variety of all samples. In addition, PNN discriminating the geographical region of samples from the same variety outperformed PNN for discriminating the region of all samples.

Mandarin is a pretty popular fruit, which is widely sold in international markets. Zhang et al. (67) developed the CSMWPCA to classify mandarin from seven geographical origins with NIR (1,000–1,800 nm). The results revealed that the second derivative was the best preprocessing method. The CSMWPCA could effectively select optimal sub-spectral regions and obtain a prediction rate of 96.61% in an independent test set.

Kim et al. (98) developed PCA and LDA models with NIR (400–4,000 cm^−1^) to discriminate five strawberry cultivars. The results based on spectra data from leaves were the same as the results based on fruits, with the accuracy of 100%.

The details (including techniques, spectral range, mode, sample numbers, feature selection/extraction methods, classification models.) of the aforementioned references related to fruits are listed in Table 2.

**Table 2:** Summary of selected references for fruits classification with visible/infrared spectroscopy.

**Sample**	**Variety/Region classification**	**Technique**	**Spectral range**	**Mode**	**Varieties/Total sample numbers**	**Extraction/Selection method**	**Model**	**References**
Grapevine	Variety	NIR	1,600–2,400 nm	Reflectance	20/544	PLS-DA	PLS-DA, ANN, SVM	(82)
Grape	Variety	NIR, ATR-MIR	400–2,500 nm 375–4,000 cm^−1^	Reflectance	2/212	PCA	LDA, PLS-DA	(90)
Grape wine	Variety	NIR	800–2,500 nm	Transmittance	2/191	No	RBFNN, LSSVM	(91)
Grape wine	Region	MIR, NIR	400–4,000 cm^−1^, 4,000–1,2800 cm^−1^	Transmission	3/540	No	PCA, SIMCA, DA	(92)
Apple	Variety	VNIR	325–1,075 nm	Reflectance	3/90	WT	BP-ANN	(49)
Apple	Variety	NIR	4,000–10,000 cm^−1^	Reflectance	4/600	MWPLSDA	KNN, PLS-DA, MWPLSDA	(93)
Apple	Variety	NIR	400–1,021 nm	Diffuse reflectance	3/300	SPA	BPNN, ELM, SVM	(94)
Apple	Variety	NIR	4,000–10,000 cm^−1^	Reflectance	4/200	PCA	FCM, PCM, GKclustering, FDCM	(76)
Apple juice	Variety	NIRMIR	400–2,498 nm 800–4,000 cm^−1^	Reflectance	4/200	No	PLS	(95)
Sugarcane	Variety	VIS/NIR	450–1,000 nm	Reflectance	4/48	No	SVM, RBFNN, KNN	(96)
Loquats	Variety& Region	NIR	800–2,500 nm	Diffuse reflectance	4/400	PCA	PNN, SIMCA	(97)
Mandarin	Region	NIR	1,000–1,800 nm	Diffuse reflectance	7/583	CSMWPCA	PCA	(67)
Strawberry	Variety	NIR	400–4,000 cm^−1^	Reflectance	5/50	LDA	PCA	(98)

### Nuts

Varieties of nuts, including almonds, pine nuts, hazelnut, and walnuts, have been studied due to their nutritional and economic value in recent years. Cortés et al. (73) compared NIR and attenuated total reflectance Fourier-transform infrared (ATR-FTIR) spectroscopy (1,000–1,700 nm) in discrimination of intact almond kernels from four Spanish varieties. Loewe et al. (36) developed discriminant partial least square (DPLS) models based on VIS/NIR (400–2,500 nm) and NIR region (1,100–2,500 nm) to classify Mediterranean pine nut from three geographical origins. The results showed that VIS/NIR contributed to achieving better performance for all cases (in-shell pine nuts, shelled pine nuts, humid flour, and dried flour) than NIR. Besides, Gu et al. (16) found the results obtained by NIR (4,000–12,000 cm^−1^) data (the mean accuracy of 99.6%) were superior to those obtained by MIR (400–4,000 cm^−1^) data (the mean accuracy of 86.6%) for discrimination of walnuts from three geographical regions.

### Meat

Alamprese et al. (99) paid attention to identifying beef meat adulteration with turkey meat in three statuses with NIR (800–2,667 nm), including fresh, frozen-thawed, and cooked. All PLS-DA models offered a high discrimination ability with the area under the curve for prediction (AUCP) over 0.920 regardless of meat in fresh, frozen-thawed, and cooked states. Besides, a binary (beef and pork) and a ternary (beef, pork, and duck) classification task was also studied with NIR (5,400–12,500 cm^−1^) (48). Furthermore, López-Maestresalas et al. (10) evaluated different adulteration levels of meat from different species (lamb, beef, pork, chicken, Lidia breed cattle and foal) with NIR (1,100–2,300 nm). The accuracy ranging from 78.95 to 100% was achieved for all the validation sets with PLS-DA. In addition to common meats, South African game meat was also studied. Dumalisile et al. (100) paid attention to game meat classification from six different species with NIR (908–1,680 nm). PLS-DA with SNV + Detrend + S-G 2nd derivative obtained the best performance, with the accuracy ranging from 70 to 96%.

Discriminating the geographical origins of meat is also important, which helps protect the international meat trade and reject the meat from the diseased area. Liu et al. (101) developed the SIMCA models with NIR (4,000–10,000 cm^−1^) to classify tilapia filets products from four geographical origins, achieving an average misclassification rate of 12.7%.

### Edible Oil

Soybean oil is one of the most important edible oil, and some genetically modified soybeans have higher oil yields and are widely accepted in the global market. However, many countries have restrictive laws regarding transgenic food importation, and they must be labeled as transgenic crops. Esteve et al. (102) employed NIR (868–1,667 nm) to discriminate conventional and genetically modified soybeans. The results indicated PLS-DA could obtain the accuracy of 98% for the classification of two soybean classes, and genetically modified soybeans trend have the moisture content than conventional soybeans.

Olive oil is widely and increasingly consumed for its nutrition and health benefits, and the quality of that strongly depends on its growing condition. Lin et al. (3) used VIS/NIR at the spectral range of 325–1,075 nm to classify olive oil from three geographical origins. The results showed that both DOSC-PLS model and DOSC-GA-PLS model had the accuracy of 97% for the external validation set, which was much higher than the PLS and GA-PLS models without DOSC.

Sesame, a significant edible oil crop, serves as an essential seasoning and material of the food industry. Choi et al. (103) measured the NIR absorbance spectrum (4,000–10,000 cm^−1^) of unprocessed sesame kernels from three different countries. The DA model obtained a total accuracy of 89.4% using the full spectra.

### Other Applications

Apart from using VIS/IR to trace the varieties and regional origins of food mentioned above, many other food products were being successfully classified with VIS/IR in recent years, including *Auricularia auricular* (104), West Lake lotus root powder (105), honey (17, 106), Chinese quince (*Chaenomeles speciosa Nakai*) (107) and sea cucumber (62). It was worth noting that Ballabio (17) employed different data fusion strategies for identification, providing a new aspect for classification tasks with NIR (4,000–7,780 cm^−1^). Guo et al. (62) developed a two-step model for classification with the second step model to further identify the wrongly classified samples or unidentified samples in the first step model, obtaining the classification accuracy of 100% for the identification of sea cucumber from nine geographical origins with NIR (800–2,500 nm). Besides, Xu et al. (105) applied the robust principal component analysis (rPCA) to detect outliers within NIR spectra (4,000-12,000 cm^−1^), which contributed to obtaining better classification results. Outliers easily occur in samples, which will lead to bias, even breakdown of the training model. Thus, applying proper algorithms to detect and expel outliers is of help for modeling. In addition to rPCA, isolation forest, on-class SVM, and elliptic envelope were also employed to detect and remove outliers within NIR spectra (4,000–12,000 cm^−1^) (4).

## Application of Hyperspectral Imaging to Trace Food Varieties and Geographical Origins

### Crop Food

Using the spectral information offered by HSI for rice varieties discrimination was widely studied and obtained pretty satisfactory results (41, 108). Kong et al. (108) found that models based on full spectra outperformed corresponding models based on optimal wavelengths for classifying four cultivars of rice with HSI (874–1,734 nm), with all accuracy over 90%. Qiu et al. (41) investigated the influence of the number (training set size 100, 200, 300, 400, 500, 600, 700, 800, 900, 1,000, 1,500, 2,000, 2,500, and 3,000) of rice samples on the performance of CNN. The results indicated that the performance of CNN improved with the increasing number of training samples. Besides, two spectral ranges (380–1,030 nm and 874–1,734 nm) were used and compared in the work.

In contrast to NIR, data fusion strategies have been studied for rice classification when using HSI for rice identification because HSI has both spectral and image information. Both Wang et al. (50) and Fabiyi et al. (20) found that the established model based on spectral-image data fusion outperformed those only based on spectral data and image data. In particular, Fabiyi et al. (20) further compared the performance of the RF model based on a combination of full spectral features and spatial features and a combination of LDA components extracted from the spectral data fused with spatial features. The results indicated integrating LDA features extracted from the spectral data and spatial features obtained better classification results on a large dataset (90 rice seed species, 96 samples per species).

Comparing to varieties and geographical origins classification of wheat with NIR spectroscopy, there were relatively fewer works focusing on wheat classification using HSI. Vresak et al. (109) and Bao et al. (110) employed HSI to discriminate twenty-seven varieties of winter wheat and five varieties of wheat seeds, respectively. Vresak et al. (109) found that the KNN model based on HSI (375–970 nm) only obtained the accuracy below 30% with the majority varieties. The reason might be the influence of a common genetic background and large surface similarity. Bao et al. (110) found that ELM based on full spectra (974–1,734 nm) was superior to ELM based on feature wavelengths, and models based on feature wavelengths extracted by RF outperformed the corresponding models based on feature wavelengths extracted by PCA loadings and SPA.

The integrated data of image features (texture features and morphological features) and spectral features within HSI (380–1,030 nm, 924–1,657 nm, and 400–1,000 nm used in different research, respectively) were utilized to discriminate varieties and origins of maize kernels in several research. All results showed a trend that the performance of the model based on data fusion was superior to that based on spectral or image features alone (1, 12, 51). To deal with increased features by integrating the spectral and image features, Huang et al. (74) adopted PCA and multidimensional scaling (MDS) to reduce fused spectral-image features to classify seventeen varieties of maize kernels. The results showed the effectiveness of feature reduction. Bai et al. (111) applied HSI (874–1,734 nm) to classify four varieties of common maize seed and four varieties of silage maize seeds. This work visualized hyperspectral images of the first six PCs of eight varieties of maize seed, which indicated that there were differences among different varieties of maize seeds. Moreover, both radial basis function neural network (RBFNN) and SVM achieved the accuracy of over 97% for both the classification of four varieties of common and silage maize seed.

Instead of establishing several different single models, Yang et al. (65) proposed a multi-model approach to discriminate 14 varieties of maize kernels with HSI (924–1,657 nm). This strategy consisted of a switch model and multiple sub-models. A switch model firstly achieved switch between different sub-models, then multiple sub-model completely identified partial categories. The results showed that the multi-model outperformed the single model. Besides, Miao et al. (55) introduced a manifold learning algorithm called t-distributed stochastic neighborhood embedding (t-SNE) into the field of hyperspectral imaging (386–1,017 nm) for four waxy maize kernels varieties discrimination. The results showed that the t-SNE model with Procrustes analysis pre-treatment obtained the accuracy of 97.5%.

The influence of calibration set size has also been investigated when using HSI (975–1,646 nm) to classify three maize varieties (19). Zhao et al. (19) evaluated the performance of RBFNN models with different calibration set sizes (sample size of 100, 200, 300, 400, 500, 600, 700, 800, 900, 1,000, 1,100, 1,200, 1,300, 1,400, 1,500, 2,000, 2,500, 3,000). The results showed that the prediction accuracy improved with the increase of the number of calibration samples, and after the number of samples reached 1,100, the prediction accuracy tended to remain stable.

Discrimination of the geographical origin of maize kernels is rarely studied. As for maize kernels, the germ side contains important information to identify different maize varieties. Conversely, the back of maize kernels is endosperm composed of starch, which is of less help to identify kernel varieties (80). The identification accuracy reached 98.2% on the germ-up dataset and 96.3% on the germ-down dataset with SVM based on HSI (1,110–2,500 nm) (1). Therefore, when collecting the spectra of maize kernels, the placement should be carefully considered.

The details of the references mentioned above related to crop food with HSI are summarized in Table 3.

**Table 3:** Summary of selected references for crop food classification with hyperspectral imaging.

**Sample**	**Variety/** **Region classification**	**Technique**	**Spectral range**	**Mode**	**Varieties/** **Total sample numbers**	**Features**		**Model**	**References**
						**Spectral/** **Image**	**Extraction/** **Selection method**		
Rice	Variety	HSI	874–1,734 nm	Reflectance	4/225	Spectral	PLS	PLS-DA, KNN, SIMCA, SVM, RF	(108)
Rice	Variety	HSI	400–1,000 nm	Reflectance	3/90	Spectral and image	PLS-DA	PCA, BPNN	(50)
Rice	Variety	HSI	380–1,030 nm and 874–1,734 nm	Reflectance	4/20,907	Spectral	No	CNN, KNN, SVM	(41)
Wheat	Variety	HSI	375–970 nm	Reflectance	36/1,080	Spectral	No	KNN, PCA	(109)
Wheat	Variety	HSI	874–1,734 nm	Reflectance	5/7,388	Spectra	PCA, SPA, RF	LDA, SVM, ELM	(110)
Maize	Variety	HSI	380–1,030 nm	Reflectance	6/330	Spectral and image	GLCM	PCA, PCA+GLCM, KPCA, KPCA+GLCM, LSSVM, BPNN	(51)
Maize	Variety	HSI	400–1,000 nm.	Reflectance	3/378	Spectral and image	GLRLM	LSSVM	(12)
Waxy maize	Variety	HSI	400–1,000 nm	Reflectance	4/600	Spectral and image	SPA, GLCM	SVM, PLS-DA	(1)
Maize	Variety	HSI	400–1,000 nm	Reflectance	17/1,632	Spectral and image	SPA, PCA, MDS	LSSVM	(74)
Waxy maize	Variety	HSI	386.7–1,016.7 nm	Reflectance	8/800	Spectral	SPA, PCA, KPCA, LLE, t-SNE	Procrustes analysis, FDA	(55)
Maize	Variety	HSI	874–,1734 nm	Reflectance	3/12,900	Spectral	PCA	RBFNN, SVM	(19)
Maize	Variety	HSI	924–1,657 nm	Diffuse reflectance	14/1,120	Spectral	JSWSA	LSSVM	(65)
Maize	Variety	HSI	874–1,734 nm	Reflectance	8/40,800	Spectral	PCA	RBFNN, SVM	(111)

### Beverage

Some algorithms have been introduced for the identification of tea varieties. Sun et al. (64) adopted iteratively retaining informative variables (IRIV) and variable iterative space shrinkage approach (VISSA) for five green tea varieties identification with HSI (431–963 nm). The two variables selection methods contributed to the improvement of classification accuracy and simplicity of the model. Wu et al. (72) introduced a deep convolutional neural network (DCNN) model to discriminate seven Chrysanthemum varieties using HSI (874–1,734 nm). The results showed that DCNN was superior to SVM and LR models, and DCNN based on full wavelengths obtained the best classification accuracy of 99.98% on the testing set, which was higher than 94.27% based on selected wavelengths.

When dealing with tea samples, a data distribution problem that can drift over time should be paid attention to. The harvesting time, storage time, stir-frying methods, and origin factor can influence the chemical composition of tea samples, which can cause a data distribution problem. Hong et al. (4) prepared tea samples of two regions from two different harvesting years, and it found both the “harvest year” and “geographical origin” factors had an impact on NIR responses. The “harvest year” factor had a higher weight on most of the original spectral variables. Therefore, when establishing a region-tracing model, eliminating the influence of the time factor will help avoid the case that the model based on samples from a specific year could not be successfully applied to predict samples from next year. Moreover, Hong et al. (112) used HSI systems covering the two spectral ranges of 380–1,030 nm (VIS/NIR) and 874–1,734 nm (NIR) to classify Longjing tea from six geographical origins. The results indicated that the PLS-DA model had better performance with VIS/NIR (accuracy of 91.98%) than PLS-DA with NIR (accuracy of 84.89%). Besides, the class value of each tea leaf was visualized, which could not be obtained through VIS/IR system.

To evaluate the feasibility of sparse methods for classification, Calvini et al. (113) used sparse variants of PCA (sPCA) and a sparse version of PLS (sPLS) to classify two cultivars of coffee with HSI (955–1,700 nm), which obtained similar results as classical PCA and PLS but with fewer variables. The models based on sparse methods were more interpretable and parsimonious. Except for new classification methods, the comparison between models based on pixel-wise spectra and object-wise spectra was discussed when using HSI. Zhang et al. (47) applied HSI (874–1,734 nm) and compared pixel-wise model and object-wise model to identify four varieties of coffee beans. Specifically, this work compared the prediction results of pixel-wise spectra by sample average-spectra-based models and prediction of sample average spectra by pixel-wise spectra based models. The result indicated the former condition achieved with the accuracy lower than 50%, but the latter achieved the accuracy of over 80%. The results suggested the preprocessing of WT and empirical mode decomposition (EMD) were suitable for pixel-wise spectra preprocessing. It should be noted that the comparison between object-wise spectra and pixel-wise spectra could be operated with an HSI system but not VIS/IR system.

### Fruits

Grape kernels or raisins have been widely studied in recent years. Zhao et al. (114) selected characteristic wavelengths from HSI (874–1,734 nm) according to the first six PCs loadings, which was helpful to obtain the accuracy of 94.3 and 88.7% for the calibration set and the prediction set with SVM for classifying grape kernels of three varieties, respectively. In addition to PCA, Zhao et al. (115) proposed the spectral feature extraction based on the waveform resolution method (SFEWR) to reduce data and extract features, which was superior to that feature reduction based on PCA in eight raisin varieties classification with HSI (900–1,700 nm). Furthermore, Feng et al. (52) evaluated the influence of raisins grade on SVM model performance for two varieties of raisins classification with HSI (874–1,734 nm). The results showed that using the object-wise spectra to predict object-wise spectra, the SVM model obtained the highest accuracy of 93.81%, and the SVM based on different raisin grades had significantly different prediction accuracy.

Except for spectral information in HSI, the feasibility of spectral indices calculated based on the spectral wavelengths for the classification of grapevine varieties was exploited. Mohsen et al. (116) extracted 32 spectral indices from the wavelengths (350–2,500 nm) to discriminate grapevine varieties with SVM and LDA. Combined with two feature selection methods (PLSR and ANOVA-PCA), all spectral indices-based models obtained an overall accuracy of 100% for both leaf level and canopy level. The results based on spectral indices were superior to the results based on optimal spectral wavelengths.

Lychee is a tasteful and nutritive subtropical to tropical fruit, and over 95% of world lychee production origin from Asia (117). Liu et al. (117) implemented HSI (400–1,000 nm) to identify three varieties of this regional fruit. The results revealed that SVM, BPNN, PLS-DA, and SIMCA obtained classification accuracy of 87.81%, 85.37%, 78.05 %, and 60.98 % for the prediction set, respectively.

To distinguish two cultivars of nectarines with a very similar appearance, Munera et al. (2) adopted HSI (450-1,040 nm) to develop the PLS-DA model. The results revealed that the average spectra of the fruits were superior to the pixel-wise spectra for the classification task. Moreover, the mean spectrum helped obtain the accuracy of 94%, and the accuracy was improved to 96% with 14 optimal wavelengths.

The feature selection/extraction methods and classification models of the aforementioned references related to fruits with HSI are summarized in Table 4.

**Table 4:** Summary of selected references for fruits classification with hyperspectral imaging.

**Sample**	**Variety/** **Region classification**	**Technique**	**Spectral range**	**Mode**	**Varieties/** **Total sample numbers**	**Features**		**Model**	**References**
						**Spectral/** **Image**	**Extraction/** **Selection method**		
Grape	Variety	HSI	874–1,734 nm	Reflectance	3/43,357	Spectral	PCA loadings	SVM	(114)
Grape	Variety	HSI	900–1,700 nm	Reflectance	8/1,200	Spectral	SFEWR, PCA	Neural network	(115)
Grape	Variety	HSI	975–1,646 nm	Reflectance	3/90	Spectral	PCA, ICA	SVM, RBFNN, KNN	(52)
Lychee	Variety	HSI	400–1,000 nm	Reflectance	3/122	Spectral	PCA	SVM, BPNN, NPLSDA, SIMCA	(117)
Nectarine	Variety	HSI	450–1,040 nm	Reflectance	2/250	Spectral	PLS coefficients	PLS-DA	(2)
Tomato	Variety& Region	HSI	950–2,500 nm	Reflectance	4/1,366	Spectral	No	PLS-DA	(33)

### Meat

Authentication of meat from different species is of significant importance for meat safety and quality control. Kamruzzaman et al. (118) implemented HSI (890–1,750 nm) to classify minced meat of pork, beef, and lamb with PLS-DA, which achieved the overall accuracy of 98.67% combined with optimal wavelengths. Jiang et al. (119) utilized two wavelength selection methods, including two-dimensional correlation spectroscopy (2D-COS) and PCA loadings, to select optimal wavelengths to detect beef adulteration with duck meat. The PLSR model based on optimal wavelengths selected from PCA loadings obtained better performance. Except for utilizing spectra information, integration of spectral and spatial information within HSI was also investigated. Garrido et al. (120) combined spectral and textural information to discriminate poultry, porcine, and fish samples. In this work, spectral and textural information was integrated using classification trees. The classification trees based on the predictions of the spectral and textural PLS-DA models were developed. Meanwhile, after projecting the spectral and textural traces of the PLS-DA models onto the latent variables, the classification trees based on the latent variables were constructed as well. Overall, the classification trees based on the predictions were much more sensitive and specific.

In general, it is a common fraud method to mix premium meat with cheaper meat, which requires the model is more specific and stable. Moreover, the degree of freshness and degree of freezing of meat may influence the performance of species identification, which are rarely studied.

### Edible Oil

Liu et al. (121) introduced the fuzzy rough set theory into the discrimination of three soybean varieties. Gaussian membership functions and triangular membership functions were proposed to select effective bands under various parameters. A post-pruning design was used to reduce the size of the subset further. The results showed that the information measure (IM) based band selection algorithm could still offer satisfactory and stable results under perturbations.

Xie et al. (59) adopted HSI to discriminate four sesame oil varieties. Based on full-spectrum and effective wavelengths selected by competitive adaptive reweighted sampling (CARS), SPA, and x-loading weights, all LDA and least-squares support vector machine (LSSVM) models have obtained the accuracy of over 80%. The models based on CARS achieved a recognition rate of 100%.

### Other Applications

Based on the spectral and spatial information, HSI could be exploited as a powerful tool for the traceability of black bean (78), honey (122), okra kernels (61), and mung beans (123). It was worth noting that Sun et al. (78) combined spectral and image features, and the optimal PLS-DA model obtained the accuracy of 98.33% for classifying black beans from three provinces. Also, Xie et al. (123) proposed the Modified gram-Schmidt (MGS) method to select effective wavelengths for classification of four mung bean varieties, based on which both ELM and LDA models obtained the prediction accuracy over 98%.

## Challenges and Future Perspectives

Visible infrared spectroscopy and hyperspectral imaging, which can be applied to analyze rice, maize kernels, fruits, vegetables, honey, meats, nuts, and edible oil, have been powerful tools in the field of variety and geographical origin identification for agricultural products and food. There was a trend that many works that the models based on a data fusion have better results than those based on spectra or image alone (1, 50, 51, 74, 78). That could benefit from integrated features generated by combining spectral feature and spatial feature.

### Challenges

However, there are some challenges to make full use of these techniques for variety and geographical origin identification of food at present: (i) Environment factors such as humidity and temperature affect spectra information collection, which put forward higher requirements for the classification under out-of-laboratory conditions. (ii) The spectra collected with NIR, MIR, and HSI contain hundreds of wavelengths, which tend to be collinear. Therefore, skills and time are required in processing the data. (iii) The calibration model based on a specified kind of sample has limited power to discriminant the different kinds of samples. To develop a more robust model, the sample preparation is supposed to include many more samples and cover more variations, including varieties, geographical origins, growth conditions, harvest years, even production processes. (iv) Models at the current stage often tend to be local, only suitable for samples from the same experiment, while for unknown samples, the results may be poor. Therefore, the universality and stability of the model should be improved, such as model transfer, and further research is in demand. (v) Large-class-number classification is more complex than traditional multi-classification for the increased data complexity and class overlapping. (vi) There were considerable researches just proposing a method and verifying its feasibility without conducting further research. This situation limits the development of practical applications.

It was found that path-variance between the probe and samples has a significant influence on the spectrum, which restricted the development of an in-line detection system of food (40). Samples from the same tree have different shapes and sizes. Consequently, distance variance always occurs because the probe of detection equipment is usually fixed. Therefore, how to prepare representative samples deserves careful consideration. Besides, spectroscopy techniques contain lots of wavelengths, and the collected data tend to be non-linear. Therefore, non-linear data processing is a challenging problem that has drawn increasing attention. Available solutions can be divided into two categories: kernel-based and manifold learning methods (55).

More samples covering more variations mentioned above are required and demanded to develop a more reliable and robust model. Sample preparation should be taken into account carefully according to what product we are about to analyse. For instance, the position of the fruits harvested from the mother plant was found to add variation to the quality (33). Different degrees of fruit maturity, non-uniform colors, and different sample zones all raise challenges for fruit classifications. Furthermore, the number of samples and the sample splitting methods are also significant for developing an effective method. It has been studied that the method to split samples into the calibration set and the validation set has an influence on the performance of the model (54). It revealed that Kennard–Stone method outperformed randomly splitting (54). Furthermore, the increase in the number of samples will empower big data processing and deep learning for tracing food varieties and geographical origins.

Multivariate calibration models are essential for classification and quantifying specific contents in food. Nevertheless, there exists a variety of specific agricultural products because of different regions and manufacturing processes. Samples from new geographical origins or varieties that are not provided in the training set tend to be unrecognized by the established model. Thus, a calibration model based on a specified kind of sample may have limited power to different samples. Besides, though there were various methods for classification tasks, some research just verified the approach's feasibility but did not conduct further research. Therefore, it is still far from practical application.

Furthermore, the large-class-number classification brings new challenges to pattern recognition due to increased data complexity and class overlapping (85). Fu et al. (85) clearly illustrated three difficulties of LCNC. They proposed that the influence of a large class number on traditional multi-class classification strategies such as one-over-one and one-over-rest needs to be investigated. Further, new approaches are in demand to overcome the difficulties in LCNC.

### Future Perspectives

Current studies showed good performances for identifying food varieties and geographical origins. Great potential for real-world applications could be foreseen. However, the studies mentioned above were mainly to explore the feasibility of the research objectives. Most of the studies lacked consistency, and further investigations lacked. Moreover, the researches covered a wide range of food types. More efforts should be made to conduct the follow-up studies to bring the researches from theory to applications.

In future studies, more attention should be paid to data analysis. For each type of food, a general data analysis flowchart could be introduced. Data analysis methods that could obtain robust and good results could be summarized and used for further studies. New data analysis strategies could also be introduced for better classification performances. The recently booming data analysis techniques, such as big data and deep learning, will significantly improve the accuracy of identifying food varieties and geographical origins. Model transfer, transfer learning, reinforcement learning, and other methods will enhance the universality and stability of models. Deep learning-based artificial intelligence will have many applications in the field of food identification. Conducting transfer learning between different applications will reduce the cost of building models for a specific application.

On the other hand, the miniaturization and portable development of spectroscopy instruments will be major trends for identifying food varieties and geographical origins as well as other food properties with the development of the microelectromechanical system (MEMS) and computer technology. At that time, computing power will increase significantly. More computing will be handed over to cloud computing. The portable device will be mainly responsible for collecting and transmitting information more efficiently. In general, advancements in processing speed of algorithms and data analysis, enhancement in image processing techniques for real-time applications for food identification, and development of low-cost imaging equipment are still of importance. Enhancing the continuity of research and achieving transfer learning between different instruments is necessary to attain food identification applications.

## Conclusion

This review summarized the application of infrared spectroscopy and hyperspectral imaging to identify food varieties and geographical origins. Various food types were studied, including common crop food, beverage, fruits, nuts, meat, edible oil, and other food types. The applications of various studies were introduced with the research objectives, analytical techniques, and results. We summarized the sample preparation, equipment settings, and data analysis strategies of the presented researches. Challenges and future perspectives of identifying food varieties and geographical origins were also discussed in detail. The presented research results illustrated the feasibility of using infrared spectroscopy and hyperspectral imaging to identify food varieties and geographical origins. However, the consistency of the researches of a specific food type should be kept. As for future studies, more efforts should be made to conduct follow-up studies to bring the researches from theory to applications. The ultimate goal for identifying food varieties and geographical origins was to bring these techniques into real-world applications. Thus, the miniaturization and portable development of spectroscopy and spectral imaging instruments should be developed for online detection at a low cost. Moreover, the utilization and development of reliable and high-performance data analysis strategies should also be conducted to establish robust models with good performances. According to this review, more work needs to be done.

## Author Contributions

LF: conceptualization, funding acquisition, writing—original draft, and writing—review and editing. BW: writing—original draft and visualization. SZ: supervision and writing—review and editing. YH: conceptualization and funding acquisition. CZ: conceptualization, investigation, software, and writing—review and editing. All authors contributed to the article and approved the submitted version.

## Conflict of Interest

The authors declare that the research was conducted in the absence of any commercial or financial relationships that could be construed as a potential conflict of interest.

## Glossary

BPNN: Back propagation neural network
BPR: Biomimetic Pattern Recognition
CARS: Competitive adaptive reweighted sampling
CNN: Convolutional neural network
CSMWPLS: Changeable size moving window partial least squares
DA: Discriminant analysis
DCNN: Deep convolution neural network
DOSC: Direct orthogonal signal correction
DPLS: Discriminant partial least squares
ELM: Extreme learning machine
EMD: Empirical mode decomposition
FCM: Fuzzy c-means clustering
FDA: Factorial discriminant analysis
FDCM clustering: Fuzzy discriminant c-means clustering
GA: Genetic algorithm
GC: Gas chromatography
GC–MS: Gas chromatography–mass-spectrometry
GK clustering: Gustafson–Kessel clustering
GLRLM: Gray-level run-length matrix analysis
HPLC: High-performance liquid chromatography
HSI: Hyperspectral imaging
ICA: Independent component analysis
IR: Infrared spectroscopy
IRIV: Iteratively retaining informative variables
iPLS: Interval partial least squares algorithm
KNN: K-nearest neighbor algorithm
KND: Karl Norris derivative filter
LE: Laplacian eigenmaps
LLE: Locally linear embedding
LPP: Locality Preserving Projection
LSSVM: Least-squares support vector machine
LTSA: Local Tangent Space Alignment
MIR: Mid-infrared spectroscopy
MSC: Multiplicative scatter correction
MLP: Multilayer perceptron
MWPLS: Moving window partial least squares
MWPLS-DA: Moving window partial least squares discriminate analysis
NIR: Near infrared spectroscopy
OLDA: Orthogonal linear discriminant analysis
OPS: The ordered predictor selection algorithm
OSC: Orthogonal signal correction
OVO: One-versus-one
OVR: One-versus-rest
PCA: Principle component analysis
PCM: Possibilistic c-means clustering
PLSCM: Partial least squares class model
PLS-DA: Partial least squares discriminate analysis
PNN: Probabilistic neural networks
PTR-MS: Proton transfer reaction-mass spectrometry
QDA: Quadratic discriminant analysis
RBFNN: Radial basis function neural network
RF: Random forest
rPCA: Robust principal component analysis
SDA: Stepwise discriminant analysis
SFDA: Stepwise forward discriminant analysis
SFEWR: Spectral feature-extraction method based on waveform resolution
S-G: Savitzky-Golay algorithm
SNV: Standard normal transformation
SPA: Successive projections algorithm
Si-PLS: Synergy interval partial least squares algorithm
SIMCA: Soft independent modeling of class analogy
SVM: Support vector machine
SVDD: Support vector data description
SVSKLPP: Supervised Virtual Sample Kernel Locality Preserving Projection
SVSLPP: Supervised Virtual Sample Locality Preserving Projection
t-SNE: T-distributed stochastic neighborhood embedding
UVE-PLS: Uninformative variable elimination based on partial least squares
VISSA: Variable iterative space shrinkage approach
WT: Wavelet transform.
